# Fabrication, In Vitro, and In Vivo Assessment of Eucalyptol-Loaded Nanoemulgel as a Novel Paradigm for Wound Healing

**DOI:** 10.3390/pharmaceutics14091971

**Published:** 2022-09-19

**Authors:** Anis Rehman, Muhammad Iqbal, Barkat A. Khan, Muhammad Khalid Khan, Bader Huwaimel, Sameer Alshehri, Ali H. Alamri, Rami M. Alzhrani, Deena M. Bukhary, Awaji Y. Safhi, Khaled M. Hosny

**Affiliations:** 1 Drug Delivery and Cosmetic Lab (DDCL), Gomal Center of Pharmaceutical Sciences, Faculty of Pharmacy, Gomal University, Dera Ismail Khan 29050, Pakistan; anisrehman700@gmail.com (A.R.); iqbalmiani@gmail.com (M.I.); barkat.khan@gu.edu.pk (B.A.K.); khalid.gomalian@gmail.com (M.K.K.); 2 Department of Pharmaceutical Chemistry, College of Pharmacy, University of Ha’il, Ha’il 81442, Saudi Arabia; b.huwaimel@uoh.edu.sa; 3 Department of Pharmaceutics and Industrial Pharmacy, College of Pharmacy, Taif University, P.O. Box 11099, Taif 21944, Saudi Arabia; s.alshehri@tu.edu.sa (S.A.); r.zhrani@tu.edu.sa (R.M.A.); 4 Department of Pharmaceutics, College of Pharmacy, King Khalid University, Abha 62529, Saudi Arabia; aamri@kku.edu.sa; 5 Department of Pharmaceutics, College of Pharmacy, Umm Al-Qura University, Makkah 21955, Saudi Arabia; dmbukhary@uqu.edu.sa; 6 Department of Pharmaceutics, College of Pharmacy, Jazan University, Jazan 45142, Saudi Arabia; asafhi@jazanu.edu.sa; 7 Department of Pharmaceutics, Faculty of Pharmacy, King Abdulaziz University, Jeddah 21589, Saudi Arabia

**Keywords:** sustainability of natural resources, eucalyptol, wound healing, topical delivery, nanoemulgel, zeta potential

## Abstract

Wounds are the most common causes of mortality all over the world. Topical drug delivery systems are more efficient in treating wounds as compared to oral delivery systems because they bypass the disadvantages of the oral route. The aim of the present study was to formulate and evaluate in vitro in vivo nanoemulgels loaded with eucalyptol for wound healing. Nanoemulsions were prepared using the solvent emulsification diffusion method by mixing an aqueous phase and an oil phase, and a nanoemulgel was then fabricated by mixing nanoemulsions with a gelling agent (Carbopol 940) in a 1:1 ratio. The nanoemulgels were evaluated regarding stability, homogeneity, pH, viscosity, Fourier-transform infrared spectroscopy (FTIR), droplet size, zeta potential, polydispersity index (PDI), spreadability, drug content, in vitro drug release, and in vivo study. The optimized formulation, F5, exhibited pH values between 5 and 6, with no significant variations at different temperatures, and acceptable homogeneity and spreadability. F5 had a droplet size of 139 ± 5.8 nm, with a low polydispersity index. FTIR studies showed the compatibility of the drug with the excipients. The drug content of F5 was 94.81%. The percentage of wound contraction of the experimental, standard, and control groups were 100% ± 0.015, 98.170% ± 0.749, and 70.846% ± 0.830, respectively. Statistically, the experimental group showed a significant difference (*p* < 0.03) from the other two groups. The results suggest that the formulated optimized dosage showed optimum stability, and it can be considered an effective wound healing alternative.

## 1. Introduction

Chronic wounds are known to greatly influence public health. For example, diabetic-related foot ulcers, which are very common, represent 50% of all diabetes-based complications and lead to a 10-year decrease in the average age of diabetic patients [1]. Retardation in the normal chain of biochemical and cellular incidents that usually contribute to restoration of skin rigidity may result in wound-healing delays. Elements that may hinder wound healing mainly include the presence of concurrent health issues (e.g., immune system diseases, diabetes, and chronic peripheral vascular disorders) and/or problems such as infectious or inflammatory diseases [2].

A series of undisturbed and sometimes overlapping events, including reepithelization, revascularization, hemostasis, reproduction, and the reconstruction of scar tissue, comprise the wound-healing process. Therefore, such chronic problems and their related healing-resistant wound complications have necessitated the evolution of novel therapeutics in the nanosized range, in an effort to support the recovery process and revive wounded tissues [3].

Plant-derived essential oils and their bioactive content employed for their antibacterial activity are thought to be safer compounds than synthetic products. Essential oils were proven to have antifungal, antibacterial, and anti-inflammatory characteristics [4]. According to the study of Infante et al. (2022), essential oils, including tea tree, lavender, and eucalyptus oils, are considered non-cytotoxic or non-phototoxic when applied to the skin, regardless of the dose [5].

Eucalyptus oil is an essential oil that is obtained from *Eucalyptus globulus* and has roughly 45.4% of 1,8-cineole (i.e., eucalyptol [Eu]). EU is a compound known to have strong antimicrobial actions against human and food-borne microbes [6,7]. The main and most important oil component is 1,8-cineole (eucalyptol: 60–85%). Due to its natural origin, 1,8-cineole is also termed eucalyptol, but it should not be confused with eucalyptus oil, which is a mixture of many other components. Eucalyptol is obtained from eucalyptus tree leaves by distillation. According to a previously reported work, *E. globulus* oil enhanced the capillary permeability and improved the wound-healing process following its administration via the intradermal route [8].

Nano delivery systems are unique due to their small particle size, surface charge, and hydrophobicity, which dramatically increase their permeability [9]. Nanoemulsions (NEs) are revolutionary delivery paradigms that are convenient for promoting parameters such as solubility and stability [10,11,12]. They are composed of oil droplets in the nano-range stabilized by a surfactant and co-surfactant, and they have been extensively investigated as drug delivery models [13]. The nanometric size of oil phase droplets was thought to be the main reason for the value of nanogels. Hence, the use of an EU nanogel could be a promising pathway for wound management [14].

Gels are comparatively recent dosage forms that are usually developed by mixing large amounts of aqueous, hydroalcoholic, or nonpolar vehicles in a three-dimensional system of polymeric materials. Gels a are comparatively novel dosage forms, capturing the attention of many researchers due to their nano size, penetration rate, and their three-dimensional structure. Gels can be easily developed by mixing large amount of aqueous, hydro-alcoholic, or non-polar vehicles [15,16]. They attract the attention of researchers owing to their great convenience, ease of application, and controlled release properties in comparison with conventional ointments and creams. Their superior controlled release properties may be the main factor in their appeal [17]. However, they were found to be less efficient in delivering hydrophobic drugs [18]. Therefore, emulgels, which combine emulsions and gels, are an acceptable alternative that enables dosage form developers to introduce hydrophobic drugs topically [19]. The integration of the emulsions with the gels makes these dosage forms very popular because they exhibit the advantages of emulsions, such as the ability to provide controlled drug release, as well as the advantages of gels, such as high thermodynamic stability [20].

Based on the aforementioned information, the objective of the current investigation was to develop and characterize nanoemulgels loaded with EU and assess their wound-healing activity in rabbits.

## 2. Materials and Methods

### 2.1. Materials

Black seed oil was purchased from Marhaba Laboratories Ltd. (Lahore, Pakistan), Propylene glycol, Tween 80, Span 60, triethanolamine, and Carbopol 940 were supplied by Sigma-Aldrich (St. Louis, MO, USA); eucalyptol was purchased from British Drug Houses Ltd. (London, UK); and Carbopol 940 and distilled water were supplied by the Research Laboratory of Gomal University (Dera Ismail Khan, Pakistan).

### 2.2. Methods

#### 2.2.1. EU-Loaded Nanoemulsion Preparation

The nanoemulgels that encapsulated EU were formulated by the previously described solvent emulsification diffusion method, with slight modifications [21]. The nanoemulgels with different quantities of ingredients were prepared in two steps. The first step was to prepare the nanoemulsion. The aqueous phase, composed of the surfactant Tween 80 and distilled water, was accurately measured on a digital scale and placed in a 100-mL beaker. The beaker was covered with aluminum foil and placed in a water bath at 70 °C for 20 min. After heating, the beaker was placed on a magnetic stirrer and the substance was mixed at 500 rpm for 30 min. For the oil phase, black seed oil, Span 60, and propylene glycol were placed in a 100-mL beaker, which was covered with aluminum foil and placed in a water bath at 70 °C for 20 min. Then the beaker was placed on the magnetic stirrer and the EU was incorporated with continuous mixing at 500 rpm for 30 min.

A coarse emulsion was initially formed by adding the oil phase drop by drop into the aqueous phase in a 500-mL beaker with continuous stirring. The coarse emulsion produced was converted to a nano-size with the help of a high-speed homogenizer (HG-15A-Set-A) at 5000 rpm for 8 min and kept overnight to remove all the bubbles and set the kinetic energy of the molecules.

#### 2.2.2. EU-Loaded Nanoemulgel Preparations

One gram of Carbopol 940 was mixed with 99 mL of distilled water in a 500-mL beaker and placed on a hot-plate magnetic stirrer at 45 °C and 500 rpm for 1 h until a clear solution was formed. Then, the nanoemulsion was added to the prepared gelling agent in a 1:1 ratio with continuous stirring at 1000 rpm for 10 min to form a nanoemulgel, and the pH was adjusted to 5–6 by adding triethanolamine. The compositions of the prepared formulations are presented in Table 1.

#### 2.2.3. In Vitro Characterization of EU-Loaded Nanoemulgels

##### Stability Studies

For stability studies, all the formulations were kept at temperatures of 8 °C, 25 °C, 40 °C, and 40 °C + 40% relative humidity (RH) for 28 days. At different time intervals (12 h, 24 h, 36 h, 48 h, 72 h, 7 d, 14 d, 21 d, and 28 d), the samples were examined for homogeneity, liquefaction, phase separation, and color change. To observe the phase separation visually, all the formulations were centrifuged at 5000 rpm and 10,000 rpm for 10 min [22].

##### Organoleptic Evaluation

All the formulations were inspected visually for homogeneity by placing them in tubes and checking their appearance for the presence of any agglomerates [23].

##### Viscosity Measurements

The viscosity of all the formulations was determined using an NDJ-5s viscometer at the previously stated time intervals with spindle No. 2 at 6, 12, 30, and 60 rpm. Each formulation was measured in triplicate, and the average values were noted [24].

##### Determination of Spreadability

The spreadability coefficient was assessed by the slip-and-drag method in which two glass slides with the same dimensions were used, with one slide fixed at a wooden block and the other placed above the fixed slide. About 1 g of each nanoemulgel was placed on the fixed slide, sandwiched between the two slides, and a solid weight of about 100 g was placed on the upper slide for 5 min to remove the entrapped air. The upper slide was attached to a pulley through a hook and thread. The spreadability was determined by hanging a weight on the thread of the upper slide, allowing it to slide, and then recording the time needed to separate the upper slide from the lower one. The procedure was repeated three times for each formulation, and the spreadability was determined using the following equation [25].

(1) $$S=\frac{M\times L}{T}$$

where S = spreadability, M = weight tied to upper slide, L = length of slide, and T = separation time.

##### Drug Content Analysis

The drug content was tested by dissolving 1 g of F5 in a standard phosphate buffer (PBS, pH 7.4) in a 100-mL flask with continuous stirring for one-half hour and adjusting the final volume to 100 mL. The obtained solution was filtered through Whatman filter paper (grade 42), and 1 mL of filtrate was diluted up to 10 mL with PBS. The same procedure was adopted for the standard sample. The absorbance of the resulting solution was measured at 556 nm using a UV visible spectrophotometer, and the content of EU was calculated using the following equation [22].

Drug concentration % = (Absorbance of sample/Absorbance of standard) × 100(2)

##### In Vitro Drug Release Evaluation

For the in vitro drug release assessment, a Franz diffusion cell was used. The cellulose acetate membrane (ADVANTEC C300A142C) was immersed in 5.5 acetate buffer solution and placed between the receptor and donor compartments. A specific amount of the nanoemulgel diluted in PBS was placed in the donor chamber. At specific time intervals, samples were taken from the receptor compartment with a syringe and analyzed with a UV visible spectrophotometer [26]. The dissolution medium was immediately replenished with equal volumes of the buffer.

##### Determination of pH

A digital pH meter was used to determine the pH of all formulations at room temperature (Table 1). After recording the results of the preceding tests, it was found that formulation F5 had the most desirable pH, as well as other features, at the different temperature intervals and humidity levels mentioned in the preceding sections [22].

##### Zeta Potential and Particle Size Determination

The Malvern Zetasizer Nano ZS was used to measure the zeta potential, mean droplet size, and polydispersity index (PDI) of the drug-loaded nanoemulsion (S1), drug-loaded nanogels (S2), and blank nanoemulsion (S3) by the dynamic laser-scattering process (Malvern Instruments, Malvern, UK). A sample of 1 mL of each solution was diluted 100 times for the measurements. Each sample was measured in triplicate [27].

##### FTIR Studies

For analyzing the desired esterification reactions and to determine the compatibility between the drug and excipients used, FTIR spectroscopy (FT-IR-4100 type A) was performed. A sample was placed on top of the clear crystal surface of the machine and a grip was placed on the sample until a click was heard. The spectra were recorded in the range of 4000 to 400 cm^−1^ at a resolution of 4 cm^−1^. Seven samples were used for FTIR analysis: 1, Tween 80; 2, Span 60; 3, propylene glycol; 4, black seed oil; 5, Eu; 6, F5 formulation; and 7, Carbopol 940. The above-mentioned method was repeated for each sample [27].

##### In Vivo Studies

*Ethical considerations*

Ethical approval was obtained from the institutional ethical review board (ERB) of Gomal University, Dera Ismail Khan, Reference no. 117/ERB/GU. All the protocols for laboratory animals, as described in the National Institutes of Health guidelines, were followed.

*Experimental animals*

For the in vivo study, 15 healthy male rabbits of a local breed weighing 1.5–1.8 kg were selected. They were maintained under standard laboratory conditions and fed with a normal diet and free access to water. Adequate ventilation, as well as proper temperature and humidity conditions, were provided. The rabbits were kept independently in wooden cages for one week prior to the in vivo studies.

*Induction of wounds*

The rabbits were shaved on the dorsal side and were properly sanitized with 70% ethanol. After sanitization, the rabbits were anesthetized with 10 mg/kg xylazine and 0 mg/kg ketamine, and an incision of 2 cm was made on the dorsal side of the rabbits using a surgical blade [28].

*Animal groups*

Experimental animals were divided into three groups (n = 5 per group):

1. The first group was treated with a blank formulation (control group).
2. The second group was treated with the test formulation F5 (experimental group).
3. The third group (standard group) was treated with the commercial wound-healing formulation Quench^®^ cream containing silver sulphadiazine, considered as a drug of choice for wound healing.

The formulations were applied to the wounded rabbits twice daily for 15 days.

*Wound-healing measurement*

Contraction in the wounds showed the wound-healing rate. Nanogels and the standard market drug were applied two times a day to both the formulation group and standard group. A Vernier caliper was used to measure the wound diameter until the wound completely healed. The percentage of wound contraction was calculated using the following equation. On every other day, the wound size was measured and data was recorded [28].

(3) $$\%\;\mathrm{wound}\;\mathrm{contraction}=\frac{\left(\text{1st}\;\mathrm{day}\;\mathrm{readings}\;-\mathrm{last}\;\mathrm{day}\;\mathrm{readings}\right)}{1\text{st}\;\mathrm{day}\;\mathrm{readings}}\times 100$$

*Statistical Analysis*

SPSS (IBM, version 20, New York, NY, USA) was used to express mean ± SD and one way ANOVA for all the collected data. A p-value less than 5% (*p <* 0.05) was considered statistically significant.

## 3. Results and Discussion

### 3.1. Temperature Swing Test and Centrifugation Study

**Figure 1:**
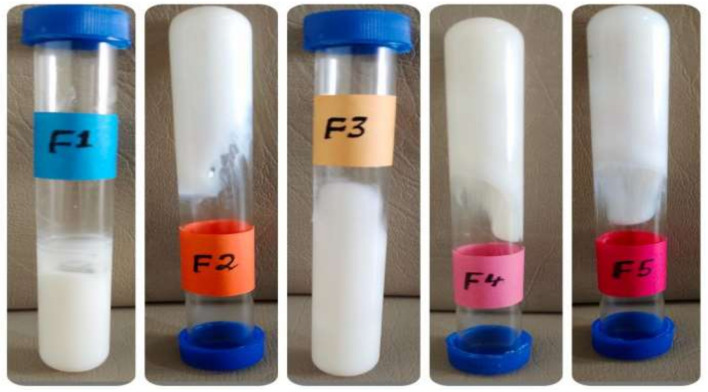
Stability of the produced formulations after centrifugation.

All the formulations were stored under different storage conditions (8 °C, 25 °C, 40 °C, 40 °C RH) for 28 days, and it was concluded that all the formulations were stable after centrifugation; no phase separation, color change, or odor change were reported (Figure 1). Although it was expected that the rate of degradation would increase with an increase in temperature, as was reported by Bachhav et al., the stability was also influenced by the polymer and drug used. Further, it was reported that the pH had a great impact on the stability of the gel formulations, since increasing the pH of the gel decreased the transparency and disturbed the structure of the formulation [29]. Therefore, the reason for the stability of all of the formulations may have been because the formulations’ pH had been adjusted by the triethanolamine in order to simulate the pH of skin. Chen et al. investigated the effect of physical and chemical variables on the consistency of Carbopol 940 and 941 gels by continuous shear rheometry, concluding that continuous shear properties were not greatly affected by centrifuging, milling, temperature cycling, or aging [30]. All of the freshly prepared formulations acquired an off-white color, and no color change was observed. All the formulations were centrifuged at 5000 rpm and 10,000 rpm, but no phase separation was observed; this might have been due to the strong intramolecular forces between Carbopol 940 as a gelling agent and Tween 80 or Span 60 as a surfactant [31].

### 3.2. Homogeneity Organoleptic Test

For the homogeneity organoleptic test, all the formulations were checked physically and visually for color changes, phase separations, consistency, and liquefaction, as shown in Table 2. All the formulations were tested under different storage conditions (8 °C, 25 °C, 40 °C, 40 °C RH) for 28 days. The formulations were evaluated physically from time to time. The freshly prepared formulations were off-white in color and had a smooth consistency. For phase separation detection, centrifugation was performed at 5000 rpm and 10,000 rpm for 10 min; no phase separation was observed. The consistency of F3 and F5 was excellent, while that of F2 and F4 was good. The homogeneity of F1, F4, and F5 was excellent, while that of F2 and F3 was good.

### 3.3. Determination of Viscosity

Viscosity greatly affects drug release and is an important physical property of topical preparations [32]. In addition, viscosity also affects various characteristics of semisolid dosage forms, including the stability, spreadability, drug release, and ease of application [33]. The lowest viscosity was observed in F1, which contained the lowest emulgent concentration, while F5 showed the highest viscosity. The viscosity of the formulations was F5 > F4 > F3 > F2 > F1. Obviously, this shows that there was a direct relationship between the emulgent concentration of the nanoemulgels and the viscosity. The higher the concentration of Tween, the greater was the viscosity, and vice versa [34]. Table 3 shows the viscosity of the different formulations.

### 3.4. Spreadability of EU-Loaded Nanoemulgels

Spreadability is one of the most important criteria for determining the therapeutic efficacy of a polymeric dosage form. The efficacy of the formulation depends upon the spreading value of the nanoemulgel, and this is the extent of the area to which the nanoemulgel can be readily spread upon application [30]. The spreadability of nanoemulgels was, in order of viscosity and time, as follows: F1 > F2 > F4 > F5 > F3 (Table 4 and Table 5). The results showed that the spreadability was inversely proportional to the viscosity, except for F3. The greater the viscosity, the smaller was the spreadability, and vice versa [35]. Several studies concluded that the shearing force and its magnitude depend upon the composition of formulation, and that the viscosity is inversely proportional to the spreadability [35,36,37]. Table 4 summarizes the mean values of the spreadability of the tested formulations.

### 3.5. Drug Content Analysis

Determining the drug content is one of the main prerequisites for any type of dosage form. As per Garala et al., the specific amount of drug in any formulation should not vary by a certain limit from the labeled amount [38]. The percentages of drug content in the EU-loaded optimized F5 nanoemulgel was 94.81%. This result revealed that the drug content remained within the official limits (100 ± 10%). This showed that the drug was uniformly distributed throughout the nanoemulgel.

### 3.6. In Vitro Drug Release Study

**Figure 2:**
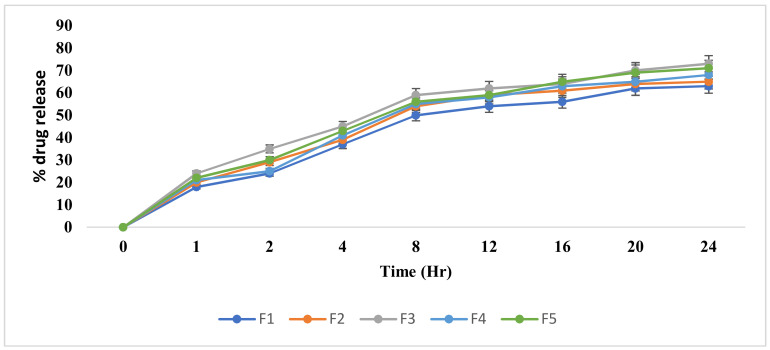
Percentage of EU release from nanoemulgel formulations. The data is presented as the mean ± SD and analyzed using one way ANOVA. *p* < 0.01 refers to F5, which is statistically significant for F1–F4.

The polymer and emulsifier affect the viscosity and drug release from the formulation. When the concentration of these increases, the formulation becomes more viscous and rigid, and this retards the release of the drug from the dosage form [39]. The release pattern was found as F3 > F5 > F4 > F2 > F1. This shows that increasing the polymer concentration decreases the drug release from the produced gels. The percentage of EU released from the formulations was 73% from F3, 71% from F5, 68% from F4, 65% from F2, and 63% from F1 after 24 h (Figure 2). Such results indicate a delayed release of EU from the developed formulations, and this might have been due to the hindrance of drug release by the gel matrix. Many studies showed that drug release decreases with an increase in the concentration of the gelling agent due to the strong intramolecular forces that can be formed between the drug and the gelling agent [40,41]. In the current study, the F5 formulation was selected for an in vivo study because the percentage of drug released from it was the best among all the formulations with regard to time. It was observed that after 18 h, a decline in the release rate occurred; this may have been due to a decrease in the drug amount in the formulation. Similar outcomes were noted by Fong Yen et al. [42]. They reported that a progressive decrease in drug release from nanoemulgels occurred with time, and this could be ascribed to a decrease in the drug amount and an opposing increase in the concentration of the eroded polymer which consequently enhanced the viscosity of the system, leading to a decrease in the drug diffusion throughout the polymeric matrix.

### 3.7. Determination of pH

The pH is an important parameter and plays a vital role in the absorption of a drug. The pH of F5 was determined at room temperature, and the results were noted in triplicate, as shown in Table 5. With the passage of time, statistically insignificant changes in the pH were observed. It is documented in the literature that the pH greatly affects the solubility and stability of formulations. The pH must be compatible to human skin pH i.e., 4.5–6. Acidic pH favors absorption, but is highly irritating to the skin, while basic pH prevents irritation, but minimizes the spreading of the dosage form, as well as absorption [37,43,44].

### 3.8. Fourier Transform Infrared Spectrophotometer

**Figure 3:**
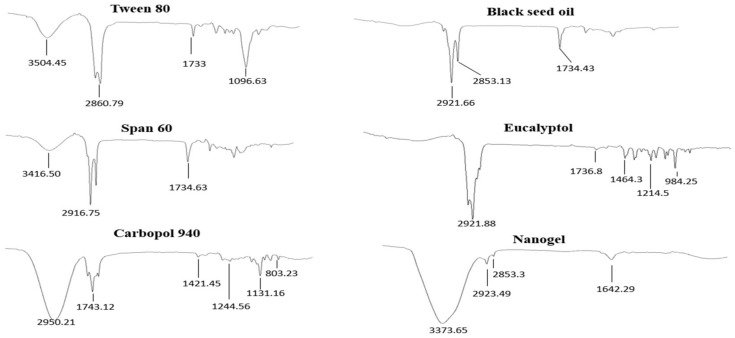
FTIR spectrum of all excipients used and the optimized F5 formulation.

The optimized nanoemulgel formulation (F5) and ingredients were characterized by ATR-FTIR (PerkinElmer 1600300, London, UK), as shown in Figure 3. ATR-FTIR spectra of the pure drug (Eu), F5 formulation, and raw materials were compared. ATR-FTIR spectra of the EU showed characteristic peaks for the C-C band at 1464.3 cm^−1^ and 1214.5 cm^−1^. Similarly, a peak at 984.25 cm^−1^ was found to be due to the stretching of the C-O-C (ether functional group). The peak of the carbonyl functional group appeared at 1736.8 cm^−1^ [45,46]. The notable characteristic peaks for black seed oil were at 2853.13 cm^−1^ (C-H in CH_2_), 2921 cm^−1^ (C-H in CH_2_), and 1734.43 cm^−1^ (C-H in HC=CH) due to the dominance of carbon chains in the fatty acids [47]. In the spectrum of the Span 60, there were peaks of the hydroxyl group at 3416.50 cm^−1^, a strong aromatic -CH_3_ group at 2916.75 cm^−1^, and a strong C=O ester bond at 1734.63 cm^−1^ [48]. Tween 80 showed many intense, sharp absorption peaks that were due to the different functional groups present in the molecules. The hydroxyl group (OH) had an absorption peak at 3504.45 cm^−1^, while the band at 2860.79 cm^−1^ was due to –CH_2_ stretching. The band at 1733 cm^−1^ can be attributed to C=O, and the band at 1096.63 cm^−1^ is due to stretching of the C–O–C. Peaks at 2923.49 cm^−1^, 2853.3 cm^−1^, and 1642.29 cm^−1^ in the nanogel formulation are related to N-H stretching, -CH_2_ group stretching, and carbonyl group stretching, respectively [49]. FTIR spectra of Carbopol 940 showed a peak in the range of 3000 to 2950 cm^−1^, representing an OH stretching vibration (i.e., O-H and intramolecular hydrogen bonding). The prominent peak between 1750 and 1700 cm^−1^ was assigned to the carbonyl C=O stretching band (i.e., C=O 0, while the peak at 1450 to 1400 cm^−1^ was assigned to C-O/O-H. The band at 1250 to 1200 cm^−1^ was assigned to the C-O-C of acrylates. The ethereal cross-linking was indicated by the prominent peak at 1160 cm^−1^, representing a stretching vibration of the C-O-C group. The band between 850 and 800 cm^−1^ indicated an out-of-plane bending of C=CH (i.e., δ=C-H) [50]. However, some changes in the peaks of the active ingredient (Eu) were observed in the nanoemulgel formulation when comparing the IR spectra of EU and F5, and this indicated that there may be some physical interactions related to the formation of weak- to medium-intensity hydrogen bonding between Carbopol 940 and the drug, but in vitro release studies showed that this type of interaction did not interfere with the release of the drug from a polymeric network.

### 3.9. Particle Size, Zeta Potential, and PDI Determination

Particle size and size distribution play a critical role in the physicochemical properties of drugs, such as the release rate, biodistribution, penetration of the skin, uptake of water and buffer by the nanosystem, and exchange of materials between the formulation and the surrounding environment [51]. Zeta analysis of the drug-loaded nanoemulsion (S1), drug-loaded nanoemulgel (F5), and blank formulations (S2) was determined (Table 6). The droplet size of S2 was 101 ± 12.6 nm, which was the smallest size as compared with S1 and F5. After loading the drug, the size of the droplet increased from 101 ± 12.6 nm to 112 ± 0.77 nm in the drug-loaded nanoemulsion (S1). Similarly, the drug-loaded nanoemulsion, when converted into a nanoemulgel (F5) by the addition of Carbopol, led to a significant increase in the droplet size (i.e., 139 ± 5.8 nm). All of the formulations had a negative charge on the surface; this is beneficial because it enhances the droplet repulsion and improves the formulation stability [52,53]. The PDI of all three formulations revealed the size distribution and homogeneity. A PDI of less than 0.45 is considered to be in the range where the dispersion is said to be homogenous [54]. The PDI ranged from 0.35 to 0.44 in the present study, showing that all of the tested formulations were homogenous.

### 3.10. In Vivo Study

**Figure 4:**
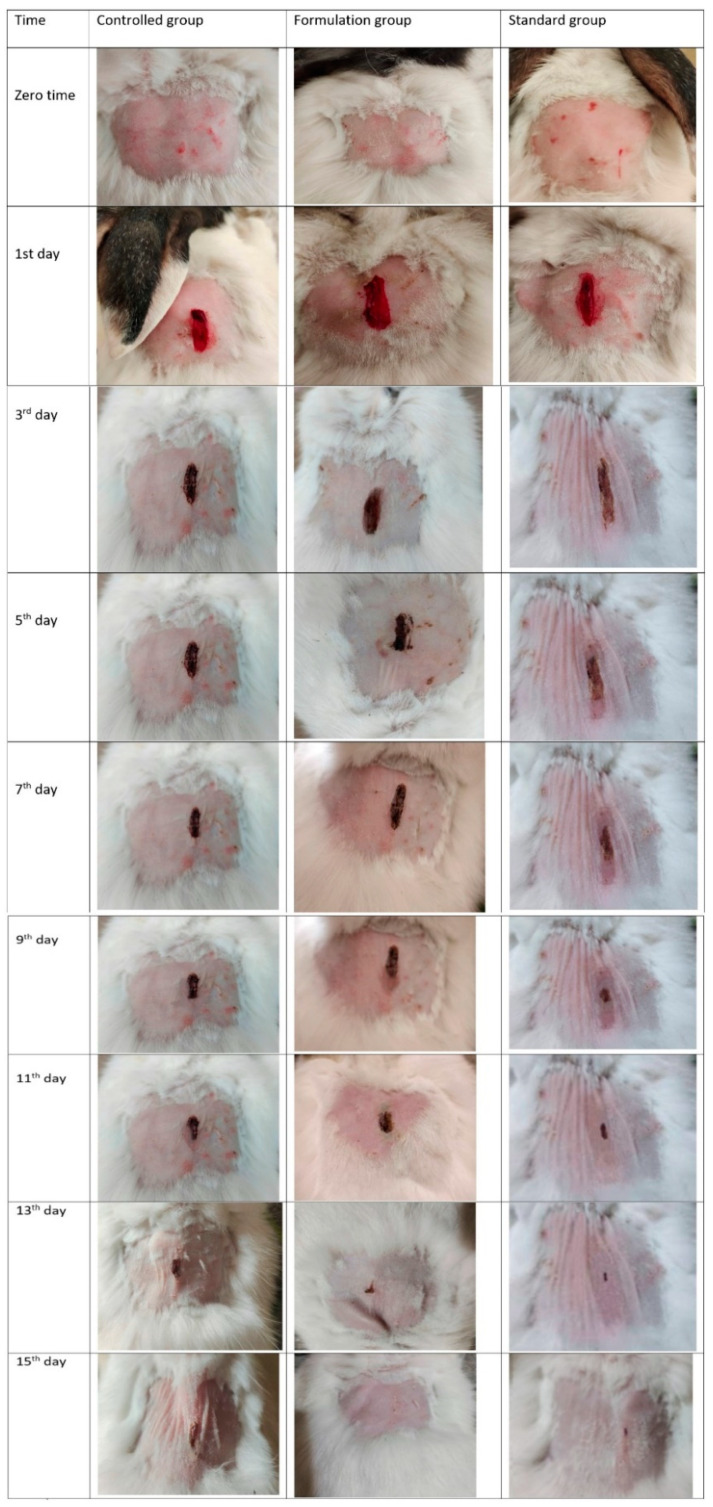
Contraction of wounds in control group, group treated with F5, and group treated with commercial product.

Topically applied drugs are more effective in wound healing due the fact that more drug is available at the injury site [55]. Hajialyani et al. stated that EU helped in accelerating wound healing because it acted as a good penetrant in transdermal and topical drug delivery systems [56]. The wound-healing assessment of F5 was carried out in rabbits for 15 days. Table 7 shows the percentage of wound contraction of the negative control, F5, and standard commercial product groups. Figure 4 illustrates the progress in the treatment of the groups. The percentage of wound contraction in the standard group was 100% on day 15, while for the negative control and F5 groups, the percentage of wound contraction observed on day 15 was 70.84% and 98.17%, respectively. The results for all three groups were analyzed by one-way ANOVA, with a level of significance of the *p*-value of less than 0.05. The statistical analysis showed that F5 had wound-healing activity similar to that of the commercial cream Quench. Pathogens play a vital role in the deterioration of a wound after injury, so it has been claimed that EU acts as a strong antibacterial agent against human and food-borne pathogens because it has good penetration and wound-healing activity [57]. The results of our research showed that F5 significantly healed the wound and had good stability under different storage conditions and temperatures. Table 7 shows the wound diameters in the animals groups during the test period.

## 4. Conclusions

From the findings of this study, it can be concluded that the nanogels were successfully prepared using a solvent emulsification diffusion method and incorporated into a gel using Carbopol 940. Thus, the current formulation has the potential to improve drug permeability and increase contact time with the skin. The current study revealed that an EU-loaded nanoemulgel is a promising approach for a topical drug delivery system that can aid wound healing.

## Figures and Tables

**Table 1:** Various compositions of nanoemulgels *w*/*w* (g).

No.	Nanoemulsion ^a^						Gelling Agent ^b^	
	Eucalyptol	Tween 80	Span-60	Propylene Glycol	Black Seed Oil	D/W	Carbopol 940	D/W
F1	8	15	7.5	13	5	51.5	1	99
F2	8	18	7.5	13	7	46.5	1	99
F3	8	20	7.5	13	8.5	43	1	99
F4	8	25	7.5	13	10	34.5	1	99
F5	8	30	7.5	13	15	26.5	1	99

^a^ and ^b^ mixed in 1:1 ratio.

**Table 2:** Physical characteristics of all formulations.

Formulation Code	Color	Phase Separation	Homogeneity	Consistency
F1	Off white	None	Excellent	Fair
F2	Off white	None	Good	Good
F3	Off white	None	Good	Excellent
F4	Off white	None	Excellent	Good
F5	Off white	None	Excellent	Excellent

**Table 3:** Viscosity of all formulations at 25 °C.

Formulation Code	Viscosity (cps)
F5	5516
F4	5180
F3	3940
F2	4380
F1	4443

**Table 4:** Spreadability values of EU-loaded nanoemulgels (mean ± SD).

Formulation Code	1st Time (s)	2nd Time (s)	3rd Time (s)	Average
F1	34.21	33.36	33.97	33.34 ± 0.48
F2	41.76	41.45	40.81	41.84 ± 0.43
F3	26.11	26.92	27.57	26.86 ± 0.73
F4	31.17	31.56	33.32	32.06 ± 0.65
F5	32.51	31.64	30.29	31.49 ± 0.84

**Table 5:** pH values of optimized F5 formulation at 8 °C, 25 °C, 40 °C, and 40 °C RH.

Time Period	8 °C	25 °C	40 °C	40 °C RH
Fresh	5.93	5.93	5.93	5.93
12 h	5.95	5.91	5.89	5.99
24 h	5.92	5.85	5.77	6.12
36 h	5.87	5.66	5.72	5.81
48 h	5.91	5.69	5.87	5.72
72 h	5.78	5.81	5.79	5.66
1 wk	5.71	5.62	5.64	5.62
2 wk	5.63	5.59	5.72	5.59
3 wk	5.61	5.54	5.69	5.50
4 wk	5.62	5.30	5.71	5.4

Note: Compared to the zero time (freshly prepared formulation), there were statistically insignificant changes observed at all four storage conditions when readings were noted at specified periods of time.

**Table 6:** Particle size, zeta potential, and PDI of S1, F5, and S2.

Formulation Code	Droplet Size (nm)	Zeta Potential (mV)	PDI
S1 (Drug loaded NE)	112 ± 0.77	−25.50	0.359
F5 (Nanoemulgels)	139 ± 5.8	−28.05	0.423
S3 (Blank NE)	101 ± 12.6	−40.5	0.446

**Table 7:** Percentage of wound contraction for the F5, standard, and control groups (mean ± SD).

Days	Control *	F5 *	Commercial Product *
3rd day	05.106% ± 0.110	25.633% ± 0.549	15.146% ± 0.254
5th day	20.050% ± 0.055	40.483% ± 0.422	30.236% ± 0.409
7th day	25.433% ± 0.388	55.536% ± 0.474	46.590% ± 0.510
9th day	40.233% ± 0.245	60.583% ± 0.514	60.326% ± 0.473
11th day	55.313% ± 0.419	80.443% ± 0.403	78.026% ± 0.380
13th day	60.420% ± 0.435	90.430% ± 0.603	89.253% ± 1.090
15th day	70.846% ± 0.830	100.000% ± 0.015	98.170% ± 0.749

* Control: formulation without Eu; F5 group: optimized formulation; commercial product: Quench Cream^®^, containing silver sulphadiazine. The data is presented as mean ± SD and analyzed using one way ANOVA. *p* < 0.02 and *p* < 0.04 refers to statistical significance of both groups from the control.

## Data Availability

Not applicable.
