# Correction: Rehman et al. Fabrication, In Vitro and In Vivo Assessment of Eucalyptol-Loaded Nanoemulgel as a Novel Paradigm for Wound Healing. *Pharmaceutics* 2022, *14,* 1971

**DOI:** 10.3390/pharmaceutics17010017

**Published:** 2024-12-26

**Authors:** Anis Rehman, Muhammad Iqbal, Barkat A. Khan, Muhammad Khalid Khan, Bader Huwaimel, Sameer Alshehri, Ali H. Alamri, Rami M. Alzhrani, Deena M. Bukhary, Awaji Y. Safhi, Khaled M. Hosny

**Affiliations:** 1 Drug Delivery and Cosmetic Lab (DDCL), Gomal Center of Pharmaceutical Sciences, Faculty of Pharmacy, Gomal University, Dera Ismail Khan 29050, Pakistan; anisrehman700@gmail.com (A.R.); iqbalmiani@gmail.com (M.I.); barkat.khan@gu.edu.pk (B.A.K.); khalid.gomalian@gmail.com (M.K.K.); 2 Department of Pharmaceutical Chemistry, College of Pharmacy, University of Ha’il, Ha’il 81442, Saudi Arabia; b.huwaimel@uoh.edu.sa; 3 Department of Pharmaceutics and Industrial Pharmacy, College of Pharmacy, Taif University, P.O. Box 11099, Taif 21944, Saudi Arabia; s.alshehri@tu.edu.sa (S.A.); aamri@kku.edu.sa (R.M.A.); 4 Department of Pharmaceutics, College of Pharmacy, King Khalid University, Abha 62529, Saudi Arabia; r.zhrani@tu.edu.sa; 5 Department of Pharmaceutics, College of Pharmacy, Umm Al-Qura University, Makkah 21955, Saudi Arabia; dmbukhary@uqu.edu.sa; 6 Department of Pharmaceutics, College of Pharmacy, Jazan University, Jazan 45142, Saudi Arabia; asafhi@jazanu.edu.sa; 7 Department of Pharmaceutics, Faculty of Pharmacy, King Abdulaziz University, Jeddah 21589, Saudi Arabia

## Error in Figure

In the original publication, there was a mistake in Figure 4 as published. In the original publication [1], there were mistakes in Figure 4. Some subfigures within Figure 4 were repeated and a switch occurred by mistake between subfigures of different groups. Some were missed and arranged in the wrong positions within other subfigures. The corrected Figure 4 appears below. The authors state that these corrections do not significantly impact the overall findings and conclusions of the paper. This correction was approved by the Academic Editor. The original publication has also been updated.

## Figures and Tables

**Figure 4:**
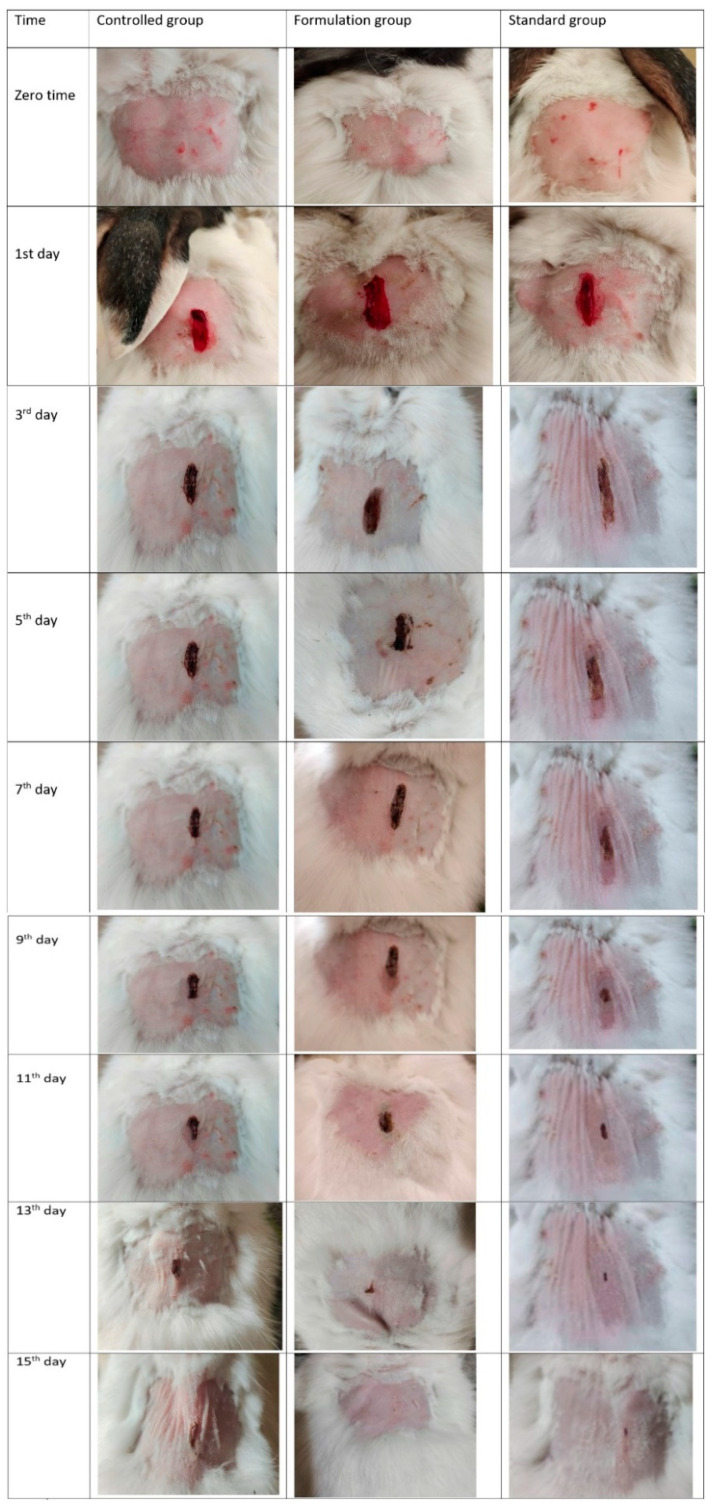
Contraction of wounds in control group, group treated with F5, and group treated with commercial product.
